# Single-cell Raman profiling of B cell differentiation and leukemic transformation with transcriptome inference

**DOI:** 10.3389/fimmu.2026.1926870

**Published:** 2026-08-27

**Authors:** Xuelian Cheng, Ming Chen, Qing Li, Linxin Dai, Jing Liu, Haoyu Wang, Yuan Zhou

**Affiliations:** 1 State Key Laboratory of Experimental Hematology, Institute of Hematology and Hospital of Blood Diseases, Haihe Laboratory of Cell Ecosystem, Institute of Hematology and Blood Diseases Hospital, Chinese Academy of Medical Sciences and Peking Union Medical College, Tianjin, China; 2 Tianjin Institutes of Health Science, Tianjin, China; 3 Department of Hematology, Nanfang Hospital, Southern Medical University, Guangzhou, Guangdong, China; 4 Fujian Provincial Key Laboratory on Hematology, Department of Hematology, Fujian Institute of Hematology, Fujian Medical University Union Hospital, Fuzhou, China; 5 Horiba Ltd., Beijing, China

**Keywords:** B cell, B-ALL, cytochrome c, Raman spectroscopy, single-cell transcriptome sequencing

## Abstract

**Introduction:**

Label-free profiling of cellular transcriptional and metabolic states during B cell differentiation and malignant transformation remains technically challenging. Raman spectroscopy offers a non-destructive alternative for single-cell biochemical characterization, yet its ability to infer transcriptomic profiles and distinguish leukemic cells has been limited.

**Methods:**

We established a Raman spectroscopy-based platform to profile B cell differentiation stages (HSC, pro-B, pre-B, naive B) and leukemic B-ALL cells at the single-cell level. Raman spectra were acquired from fixed cells and analyzed using principal component-linear discriminant analysis (PC-LDA) classification. An adversarial autoencoder (AAE) framework was employed to align Raman measurements with reference single-cell RNA-seq data, generating Raman-inferred transcriptomic profiles in the reference expression space. Cytochrome c expression was validated by flow cytometry, and metabolic pathways were examined via GSEA.

**Results:**

PC-LDA resolved four B cell differentiation stages with 96.48% accuracy, identifying Raman features consistent with cytochrome c as potential spectral markers of differentiation status, validated by flow cytometry and mitochondrial membrane potential measurements. The AAE-based model generated Raman-inferred profiles that preserved major cell-type-associated transcriptomic patterns, with a mean classification accuracy of 91.78% ± 8.13% across repeated partitions. Performance varied considerably across repeated splits for pro-B (52.48-100%) and pre-B cells (34.4-100%), indicating that distinguishing closely related stages remains challenging. Applied to B-ALL, the method distinguished cord-blood-derived normal B cells from bone-marrow B-ALL cells (96.62% accuracy) and revealed reprogramming of glucose metabolism, consistent with transcriptomic enrichment analysis.

**Conclusions:**

This study presents a proof-of-concept framework demonstrating that Raman spectroscopy, integrated with machine learning and transcriptomic alignment, enables non-destructive, fixed-cell-based omic profiling of B cell development and leukemia. The approach bridges label-free optical readouts with transcriptome-informed profiling, opening new avenues for hematopoietic research, analysis of archived specimens, and future clinical exploration. However, these findings are based on a limited number of donors and unmatched tissue sources, and require validation in larger cohorts before clinical translation.

## 1 Introduction

The biology of a cell is governed by a dynamic balance between intrinsic genetic programs and extrinsic environmental inputs (1), including differentiation cues, cytokine stimulation, and oncogenic transformation. Cells respond to environmental changes by activating gene transcription, translating mRNA into proteins, producing metabolites, and ultimately enabling physiological adaptive responses (2, 3). Single-cell omics methods have revolutionized biomedical research by capturing cellular heterogeneity at unprecedented resolution across the genome, epigenome, transcriptome, proteome, and metabolome. However, single-cell RNA-sequencing (scRNA-seq) and other profiling assays are expensive, time-consuming, require complex sample preparation, and are inherently destructive, precluding longitudinal studies of living cells and dynamic biological processes (1, 4–6).

Raman spectroscopy has emerged as a powerful label-free alternative that measures the inelastic energy shift of scattered photons caused by molecular bond vibrations (1, 7, 8). Certain biomolecules exhibit prominent, distinguishable Raman spectral peaks, enabling estimation of their abundance from signal intensities. For example, cytochrome c, lipids, and nucleic acids can be semi-quantified based on characteristic peaks at 750, 2850, and 785 cm^-1^, respectively (9–11). Alternatively, the entire Raman spectrum can be analyzed as high-dimensional data and linked to cell types and phenotypes through dimensionality reduction and classification algorithms. Principal component analysis (PCA) and orthogonal partial least squares discriminant analysis (OPLS-DA) have successfully distinguished various leukemia subtypes (8, 12, 13). With the availability of large Raman spectral datasets, more sophisticated machine learning methods, including support vector machines and random forests, can be employed to learn robust correspondences between spectral fingerprints and cell types (1, 4, 14).

Raman spectroscopy has found diverse applications in the study of leukemia (11, 12, 15–18). Efforts have ranged from characterizing metabolic reprogramming in leukemia subtypes arising from specific genetic aberrations, to developing advanced diagnostic platforms for accurate subtype identification. Our teams established a stimulated Raman platform that distinguishes six leukemia subtypes with 88.21% accuracy via label-free single-cell profiling (11). Nowakowska et al. demonstrated that Raman spectroscopy can distinguish hyperdiploid B-ALL from normal B cells and other molecular subtypes, including TCF3-PBX1, KMT2A-r, BCR-ABL1, and ETV6-RUNX1 (18). Zhang et al. highlighted that PCA-LDA models can distinguish T cells from B cells with 97.08% accuracy, while convolutional neural networks achieve 98.92% accuracy in monitoring dendritic cell maturation, underscoring the broad applicability of Raman-based approaches in immunology (15).

Cellular Raman spectra have shown considerable potential for phenotype classification and disease diagnosis. Nevertheless, the reliability of classifications depends on a clear understanding of their underlying molecular origins. This raises the question of whether Raman spectra can be used to infer scRNA-seq and other omics profiles. Specifically, this study aimed to: (i) investigate whether Raman spectra can distinguish B-cell differentiation stages; (ii) evaluate cross-modal mapping between Raman measurements and an external scRNA-seq reference using an AAE-based framework, where Raman and scRNA-seq data were not acquired from the same cells; and (iii) compare Raman profiles between normal cord-blood-derived B cells and bone-marrow-derived B-ALL cells. Collectively, this work demonstrates Raman-based transcriptomic inference in B cell development and leukemia, establishing a label-free approach for probing transcriptional and metabolic programs in fixed hematopoietic cells, with potential applications in archived specimen analysis and future clinical exploration.

## 2 Methods and materials

### 2.1 Human samples and cell preparation

The study was approved by the institutional Ethics Committee (NSFC2022035-EC-2) of the Institute of Hematology and Blood Diseases Hospital, and written informed consent was obtained from all participants. Three normal cord blood (CB) and three B-ALL bone marrow samples were obtained from the Institute of Hematology and Blood Diseases Hospital. The three cord blood samples were obtained from healthy mothers aged between 20 and 40 years, with no underlying diseases. All newborns were full-term and had no identified hematological abnormalities. Information on the three B-ALL bone marrow donors is shown in **Supplementary Table 1**, blast percentages exceeding 95% in all cases. Leukocytes were isolated using a lymphocyte separation solution (TBD LTS1077) following standard density gradient centrifugation methods. Frozen cells were quickly thawed and stained with human antibodies. DAPI (Sigma-Aldrich) was then added to the cell suspensions to exclude dead cells.

Based on the phenotypic information reported, we sorted four populations of cells from CB samples using FACS (FACSAria III, BD Bioscience): HSC (Lin^-^CD34^+^CD38^-^CD45RA^-^CD90^+^CD49f^+^CD10^-^), pro-B (CD34^+^CD10^+^CD19^+^IgM^-^), pre-B (CD34^-^CD10^+^CD19^+^IgM^-^) and naive B (CD19^+^CD24^int^CD38^int^) (19, 20). B cells (CD19^+^) were sorted from B-ALL samples. The antibodies used for sorting HSCs and B cells are summarized as follows: Lin (BV510, BD Biosciences), CD34 (APC, BD Biosciences), CD38 (PE/Cy7, BD Biosciences), CD45RA (FITC, BD Biosciences), CD90 (PerCP/Cy5.5, BD Biosciences), CD49f (PE, BD Biosciences), CD10 (APC/H7, BD Biosciences), CD34 (FITC, Biolegend), CD19 (PerCP-Cy5.5, Biolegend), IgM (Alexa Fluor 647, Biolegend), and CD24 (PE, clone ML5, Biolegend). Post-sort purity was assessed by flow cytometry immediately after sorting for each population.

### 2.2 Raman data acquisition

HSC, pro-B, pre-B, naive B, and B-ALL cells were sorted and fixed in 1% vol/vol formaldehyde for 10 min, then washed three times with deionized water. Before Raman measurements, the fixed cells were prepared by placing a 20 μL drop of cell suspension onto quartz slides by centrifugation. Single cell Raman spectra (SCRS) were obtained using an XploRA Plus Raman microscope (HORIBA) with a 532 nm laser and a ×50 objective. The laser power was approximately 10 mW, with a working spot size of ~2 μm. The spectrometer was calibrated daily using a silicon standard (520.7 cm^-1^). Spectra with signal-to-noise ratio <10 were excluded. Target cells were selected based on morphological integrity and absence of aggregation. SCRS were acquired from 600 to 1800 cm^-1^ for 1 s with a 1200 g/mm diffraction grating. Five points were measured per cell, with at least 20 cells per replicate, yielding 1,957 SCRS from 393 cells. Preprocessing was performed using Labspec6: cosmic spikes were removed using a consistent criterion (peaks >3× local median, FWHM <5 cm^-1^ replaced by interpolation); Spectra were smoothed using a Savitzky–Golay filter (window size: 7 points, polynomial order: 2); background was subtracted; and a multipoint baseline correction was applied. SCRS were normalized by unit vector normalization. Acquisition was not formally blinded, as cell types were morphologically distinguishable. However, preprocessing and statistical analysis were performed by a researcher blinded to cell-type information.

### 2.3 Cytochrome c and mitochondrial membrane potential determination

Hematopoietic stem and progenitor cells (HSPCs) and B cells were isolated from CB using Lin^-^CD34^+^ and CD19^+^ markers according to the manufacturer’s instructions. Cytochrome c and MMP were determined by flow cytometry. For cytochrome c detection, the cells were adjusted to 1×10^6^ cells/ml, and the antibody combinations were added to the corresponding cell tubes, which were incubated at 4 °C for 30 min, then the cells were treated with BD Cytofix/Cytoperm™ Fixation/permeabilization Kit and incubated with Alexa Fluor^®^ 647 anti-cytochrome c antibody (6H2.B4, Cell Signaling Technology) for 30 min at room temperature. Cells were washed two times with PBS and analyzed by flow cytometry. For MMP determination, the cells were adjusted to 1×10^6^ cells/ml, the antibodies were added to the corresponding cell tubes, which were incubated at 4 °C for 30 min, then the cells were stained with 500 μl Mito-Tracker Red CMCRos working solution (30 nM) at 37 °C for 20 min. After washing two times with PBS, the cells were analyzed by flow cytometry. All flow cytometry data were collected by using a BD Arria III flow cytometer (BD Biosciences, USA), FlowJo™ V10.6.1 (BD Biosciences, USA) software was used to analyze the flow chart and mean fluorescence intensity.

### 2.4 Principal component-linear discriminant analysis

Principal component-linear discriminant analysis (PC-LDA) was performed to classify Raman spectra from different cell populations. Raman spectra were standardized and partitioned into training and test sets (7:3) using a cell-level grouped split strategy, ensuring that all spectra from a given cell were assigned to the same dataset partition to avoid information leakage from repeated measurements. To further prevent data leakage, PCA was fitted using the training spectra only; the fitted PCA transformation was then applied to the held-out test spectra. The number of principal components (PCs) retained for LDA was determined using a pre-specified 98% cumulative explained-variance threshold, selecting the smallest number of PCs that reached this threshold within each training split. This procedure retained 95 PCs for the normal B-cell 70:30 cell-grouped split and 153 PCs for the combined normal/B-ALL 70:30 cell-grouped split. For repeated splits and cross-validation analyses, the number of PCs was recalculated within each training fold. Classification performance was assessed using confusion matrices, receiver operating characteristic (ROC) curves and the corresponding area under the curve (AUC). UMAP was used for visualization of clustering patterns.

### 2.5 Single-cell transcriptome analysis

Single-cell transcriptome data of HSC and B progenitor cells were obtained from NCBI GEO (GSE137864 and GSE149938) (19, 20). These two datasets were selected because GSE137864 provides transcriptomic data for HSC cells, while GSE149938 provides data for pro-B, pre-B, and naive B cells, together covering the four differentiation stages. Single-cell RNA-seq data were processed and analyzed using the Scanpy (https://scanpy.readthedocs.io/en/stable/index.html). Cells from different developmental stages were integrated, normalized, clustered and visualized by dimensionality reduction. Differentially expressed genes between cell populations were identified for downstream functional analyses. The genes associated with metabolic processes were downloaded from KEGG website. Metascape was used to provide a comprehensive gene list annotation and analysis resource for experimental biologists (21). R program was used to plot heatmaps (by heatmap package) and boxplots (by ggplot2 package). Detailed preprocessing information, model configuration, random seeds, and software versions are provided in the github repository (https://github.com/Bio-MingChen/Raman2SC-AAE).

### 2.6 Adversarial autoencoder-based Raman-to-transcriptome transfer

An AAE model (1) was used to transfer single-cell Raman measurements into a reference scRNA-seq expression space. Four cell types (HSC, pro-B, pre-B and naive B cells) were included in the analysis. For the reference dataset, the top 2,000 highly variable genes were selected and used for transcriptomic modeling. To avoid information leakage arising from repeated Raman measurements from the same cell, Raman spectra were partitioned using a cell-level grouped split strategy, ensuring that all spectra from a given cell were assigned to the same training or test subset. Raman data were divided into training and test sets at a 7:3 ratio based on cell identity.

An autoencoder was first trained on the reference scRNA-seq dataset to learn a transcriptomic latent space. Raman spectra were subsequently aligned to this latent space through adversarial training, allowing transcriptome-like profiles to be inferred from Raman measurements. Transferred Raman-to-scRNA profiles were evaluated using cell-type classification, low-dimensional visualization, gene-expression correlation and differential-expression analyses. The AAE used a 128-dimensional latent space with fully connected encoder and decoder networks. Raman training combined reconstruction, adversarial alignment, and auxiliary cell-type classification losses. The reference autoencoder, cell-type classifier, and Raman autoencoder were trained with fixed hyperparameters and repeated grouped partitions as described in the github repository (https://github.com/Bio-MingChen/Raman2SC-AAE).

### 2.7 Gene set enrichment analysis

A targeted gene set enrichment analysis was performed on two transcriptomic datasets (GSE48558: 27 B-ALL vs. 11 normal B; GSE116229: 8 B-ALL vs. 3 normal B) using Gene Set Enrichment Analysis (GSEA) software (v4.3.3). Based on the elevated Raman signal at 920 cm^−1^ in B-ALL cells, two metabolism-related gene sets were selected after completion of Raman analysis but prior to GSEA: HALLMARK_GLYCOLYSIS and GOBP_AEROBIC_RESPIRATION. A total of 1,000 gene set permutations were performed. The FDR was used for multiple-testing correction, with significance defined as FDR q < 0.25; nominal p values were reported descriptively only. Normalized enrichment scores (NES), nominal p values, and FDR q values are reported. This analysis was restricted to the two targeted gene sets; no unbiased gene-set screening was performed.

### 2.8 Statistical analysis

Raman spectral band intensities were compared using a linear mixed-effects model with cell population as a fixed effect and donor as a random effect, with cell-level nesting included where appropriate. Variance components were estimated by maximum likelihood using statsmodels MixedLM. Pairwise comparisons of estimated marginal means were FDR-adjusted (Benjamini–Hochberg) within each band. Significance was defined as *P < 0.05, **P < 0.01, ***P < 0.001. Full results, including marginal means, model diagnostics, and pairwise comparisons, are provided in **Supplementary Table 2**. For classification performance evaluation (PC-LDA and AAE), cell-level grouped splits were used to avoid information leakage from repeated measurements of the same cell, with spectra from the same cell assigned to the same dataset partition. Donor-level aggregation analyses were performed as supplementary validation.

## 3 Results

### 3.1 Spectro-phenotyping of hematopoietic cells at different stages

**Figure 1:**
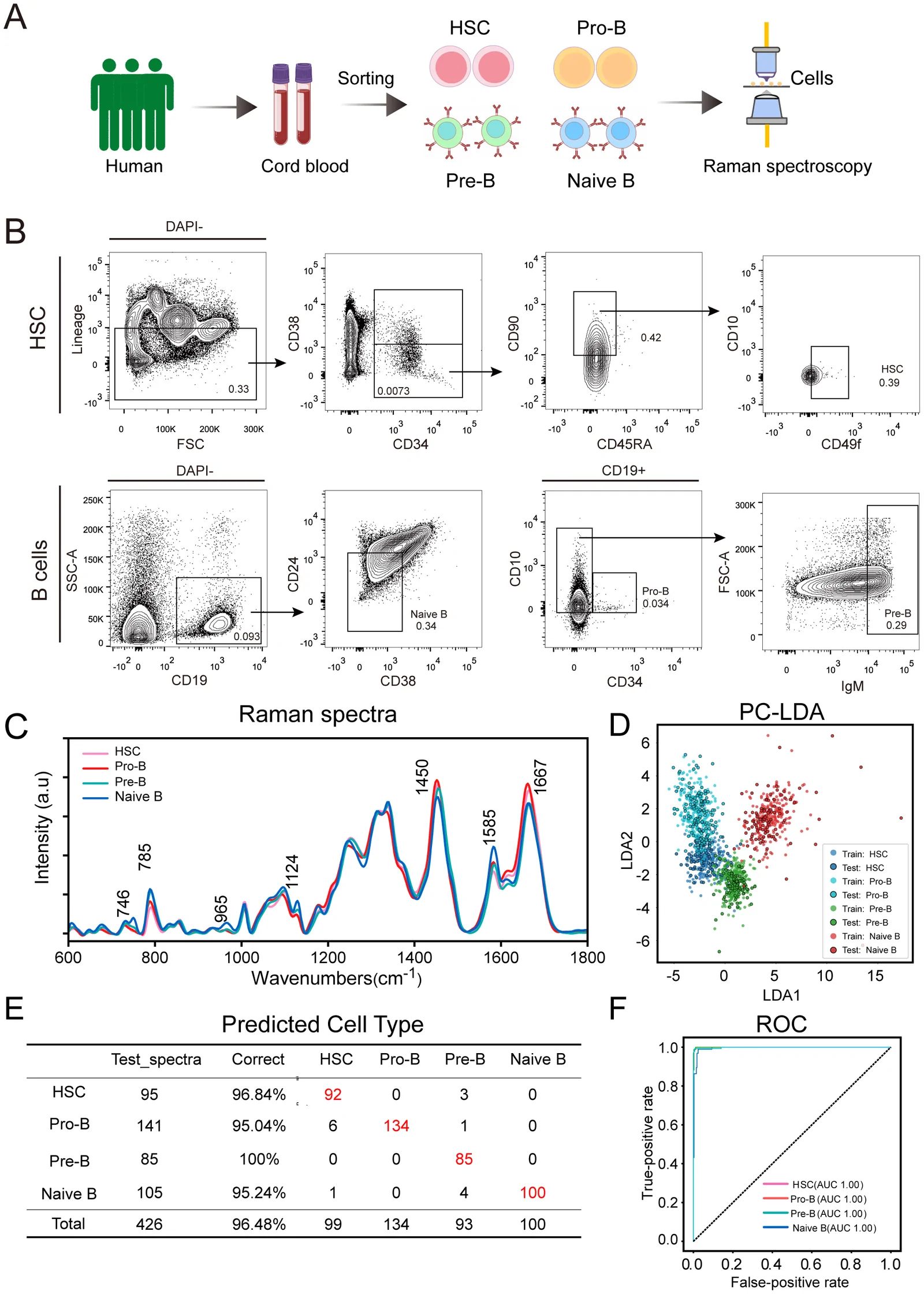
B cell differentiation monitored with Raman spectroscopy. **(A)** Schematic overview of the experimental workflow. Four stages of B cell differentiation of hematopoietic stem cells (HSCs), pro-B, pre-B, and naive B cells were isolated from human cord blood and analyzed by Raman spectroscopy. **(B)** Flow cytometry gating strategy for sorting HSCs (Lin^-^CD34^+^CD38^-^CD45RA^-^CD90^+^CD49f^+^CD10^-^), pro-B cells (CD34^+^CD10^+^CD19^+^IgM^-^), pre-B cells (CD34^-^CD10^+^CD19^+^IgM^-^), naive B cells (CD19^+^CD24^int^CD38^int^). **(C)** Representative Raman spectra showing average spectral profiles for the four populations. Characteristic peak positions are labeled. **(D)** Principal component analysis combined with linear discriminant analysis (PC-LDA) showing distinct clustering of the four populations based on Raman spectral features, indicating successful discrimination between the four developmental stages. Data points without black circles represent the training dataset, while those with black circles represent the test dataset. **(E)** Confusion matrix for the predicted classification of the PC-LDA model for test spectra of B cells at different stages. **(F)** ROC curves for the predicted classification of the PC-LDA model for B cells at different stages. n = 329 spectra for HSC, 330 for pro-B, 431 for pre-B, and 325 for naive B cells (3 donors per group).

During the differentiation process, the cells synthesized new biomolecules required to perform new functions and degrade unwanted biomolecules (22). The experimental workflow of cells at different stages, including HSC, pro-B, pre-B, and naive B cells, was shown in **Figure 1A**. The sorting strategy diagram was presented in **Figure 1B** and **Supplementary Figure 1**. Representative post-sort purity plots for each population are shown in **Supplementary Figure 2**. After smoothing, baseline correction, and normalization, the spectra were obtained (**Supplementary Figures 3A–C**). In total, 283 single cells (1415 spectra) were profiled to construct the Raman atlas. The averaged Raman spectra of each subtype were shown in **Figure 1C**. These global features were common among various cell types, including mammalian cells, reflecting the basic chemical composition of cells (4, 23). The Raman bands of 746, 785, 965, 1124, 1457, 1585, and 1667 cm^-1^ played a key role in separating four cell populations, which contained cytochrome c (746, 1124 and 1585 cm^-1^) (24–26), nucleic acids (785, 965 and 1585 cm^-1^) (24) and protein/lipids (1457 and 1667 cm^-1^) (24). Notably, while the 1585 cm^-1^ band is commonly used as a marker for cytochrome c, it is not exclusively specific to this molecule, as it also receives contributions from nucleic acids.

We next asked whether these spectra could be classified according to differentiation state using PC-LDA. Each Raman spectrum can be represented as a single point in a 987-dimensional space, where each dimension corresponds to the signal intensity at a specific wavenumber. Our results revealed that spectra from the same differentiation state formed distinct clusters in the dimension-reduced Raman space, demonstrating a strong capacity to discriminate among cell populations (**Supplementary Figures 4A, B**). For visualization, a three-dimensional PC-LDA plot and a Uniform Manifold Approximation and Projection (UMAP) plot are shown in **Supplementary Figures 4C, D**. The Raman dataset was split into a training set (70%) and a test set (30%) using cell-level grouped partitioning. The model trained on the training set achieved excellent separation of the four cell populations in a two-dimensional discriminant space (**Figure 1D**). Classification performance was rigorously validated on the held-out test set using a confusion matrix (**Figure 1E**), yielding an overall accuracy of 96.48% across 426 SCRS, with per-class accuracies of 96.84% for HSC, 95.04% for pro-B, 100% for pre-B, and 95.24% for naive B. ROC curve analysis on the test set (**Figure 1F**) further confirmed the model’s high reliability, achieving a perfect AUC of 1.00 for all four B-cell subtypes.

To assess the generalizability of the PC-LDA model to unseen donors, we performed leave-one-donor-out cross-validation using the three cord-blood donors. The model achieved an overall accuracy of 89.82%, balanced accuracy of 89.73%, and macro F1 of 89.57% across 1,415 held-out spectra (**Supplementary Table 3**). Fold-level accuracies were 76.05%, 94.80%, and 97.72% across the three donors, with the lower accuracy observed for the CB_1 donor suggesting donor-specific variability that warrants future investigation with larger cohorts.

### 3.2 Raman characterization and validation of B cell differences

**Figure 2:**
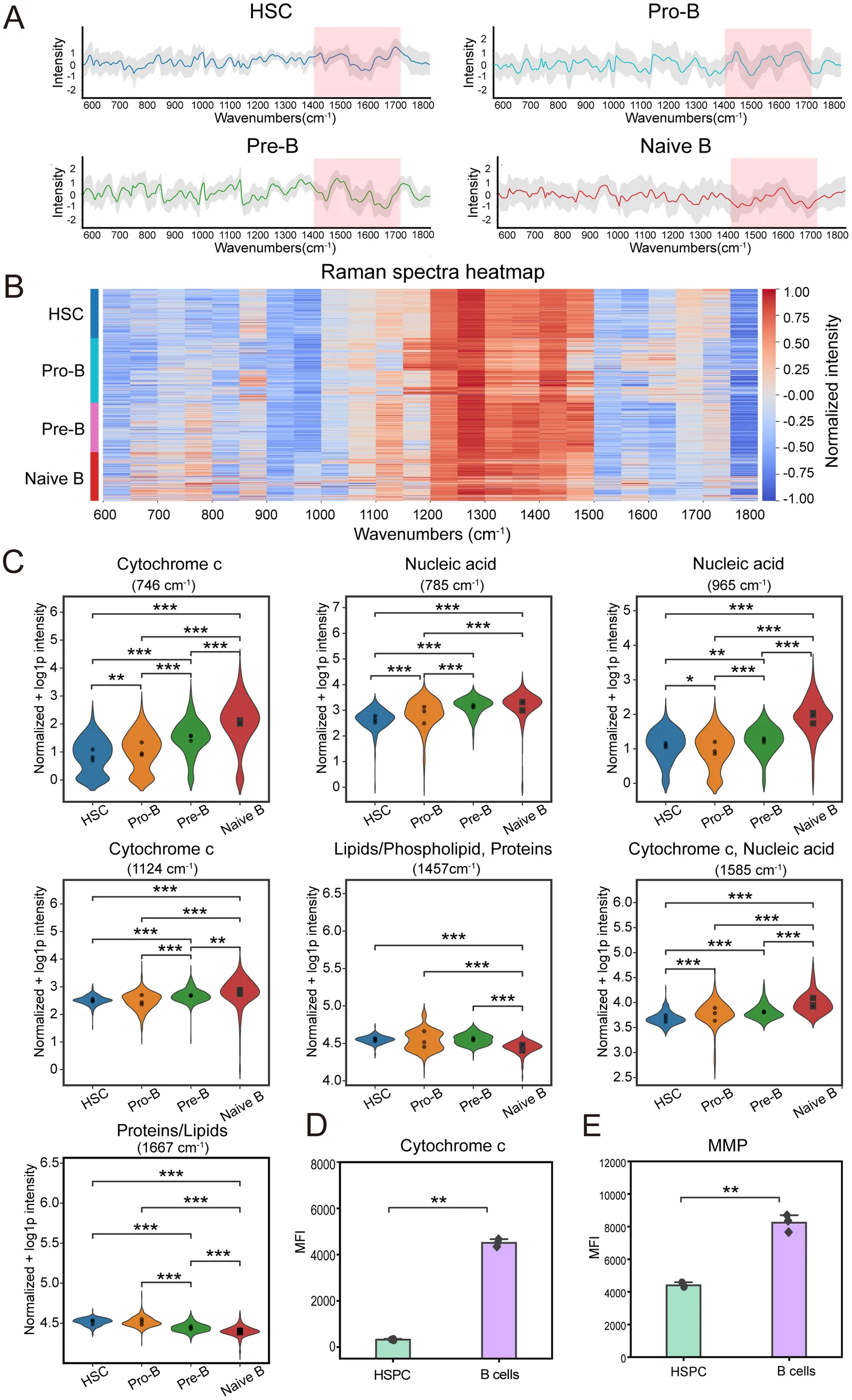
Raman spectroscopy analysis reveals differences in B cells at distinct differentiation stages. **(A)** Raman spectra of B cells at four differentiation stages, normalized to a maximum of 1 and scaled to zero mean and unit variance. **(B)** Heatmap of scaled Raman intensity across B cell subgroups. **(C)** Quantitative comparison of Raman band intensities for cytochrome c (746 cm^-1^), nucleic acid (785 cm^-1^ and 965 cm^-1^), cytochrome c (1124 cm^-1^), lipids/phospholipids and proteins (1457 cm^-1^), nucleic acid (1585 cm^-1^), and proteins/lipids (1667 cm^-1^). **(D, E)** Cytochrome c and MMP expression at broadly defined HSPCs (Lin^-^CD34^+^) and total B cells (CD19^+^) populations, measured by flow cytometry. All data are presented as mean ± standard error of the mean (SEM). Statistical comparisons between groups were performed using a linear mixed-effects model with cell population as a fixed effect and donor and cell (nested within donor) as random effects, accounting for the non-independence of repeated spectra from the same cell. Pairwise comparisons of estimated marginal means were FDR-adjusted (Benjamini–Hochberg). *P < 0.05; **P < 0.01; ***P < 0.001.

The nature of cell differentiation implies that energetic requirements and cellular morphology are altered, suggesting that cellular differentiation is accompanied by metabolic reprogramming (27, 28). During the progression of B cell differentiation, several changes in Raman bands for major cellular components and metabolites were observed. To enable a clearer comparison of the spectral differences among different cell types, the scaled Raman average spectra are presented in **Figure 2A**. The red shaded areas (1400–1700 cm^-1^) are the regions with the most pronounced differences. The heatmap in **Figure 2B** visualizes the scaled Raman intensity (1100–1500 cm^-1^), showing that the four cell types highly express these features, but with no significant differences among them. Furthermore, in the 650–900 cm^-1^ range, a clear trend was observed: Raman intensity increases with the extent of B cell differentiation. To confirm the variations in biomolecules at different cell stages, we further performed semi-quantification of biomolecules by integrating relevant Raman band areas. As shown in **Figure 2C**, the Raman signal intensities at bands consistent with cytochrome c (746, 1124, and 1585 cm^-1^) increased significantly during cellular differentiation. Nucleic acids bands exhibited a similar trend to cytochrome c (785, 965 and 1585 cm^-1^). However, proteins and lipids did not show a significant trend with B cell differentiation. The analysis also indicated that the spectral features associated with protein synthesis did not correlate with the observed changes in DNA/RNA-related bands. To evaluate the impact of inter-donor variability on these observations, we performed donor-level aggregation analyses by calculating the mean spectral intensity at each band per donor prior to group comparisons. The trends observed at the donor level were broadly consistent with the spectrum-level results, supporting the robustness of our findings (**Supplementary Figure 5**).

Next, we evaluated the content of cytochrome c using flow cytometry. The cytochrome c level in broadly defined HSPCs (Lin^-^CD34^+^) was lower than that in total B cell (CD19^+^) populations (**Figure 2D**), indicating that the content of cytochrome c increased remarkably upon differentiation at the protein level across the two extremes of the differentiation axis. To study the relationship between cytochrome c and mitochondrial function in B cells at different stages, MMP was further examined. During B cell differentiation, the MMP of B cell populations was higher than that of HSPCs (**Figure 2E**), indicating an increase in MMP during differentiation. Collectively, these results demonstrate that Raman spectroscopy can capture stage-specific biomolecular changes during B cell differentiation, with cytochrome c identified as a potential spectral marker that correlates with both metabolic activation and mitochondrial remodeling.

### 3.3 Stage-specific transcriptomic profiles of hematopoietic cells

**Figure 3:**
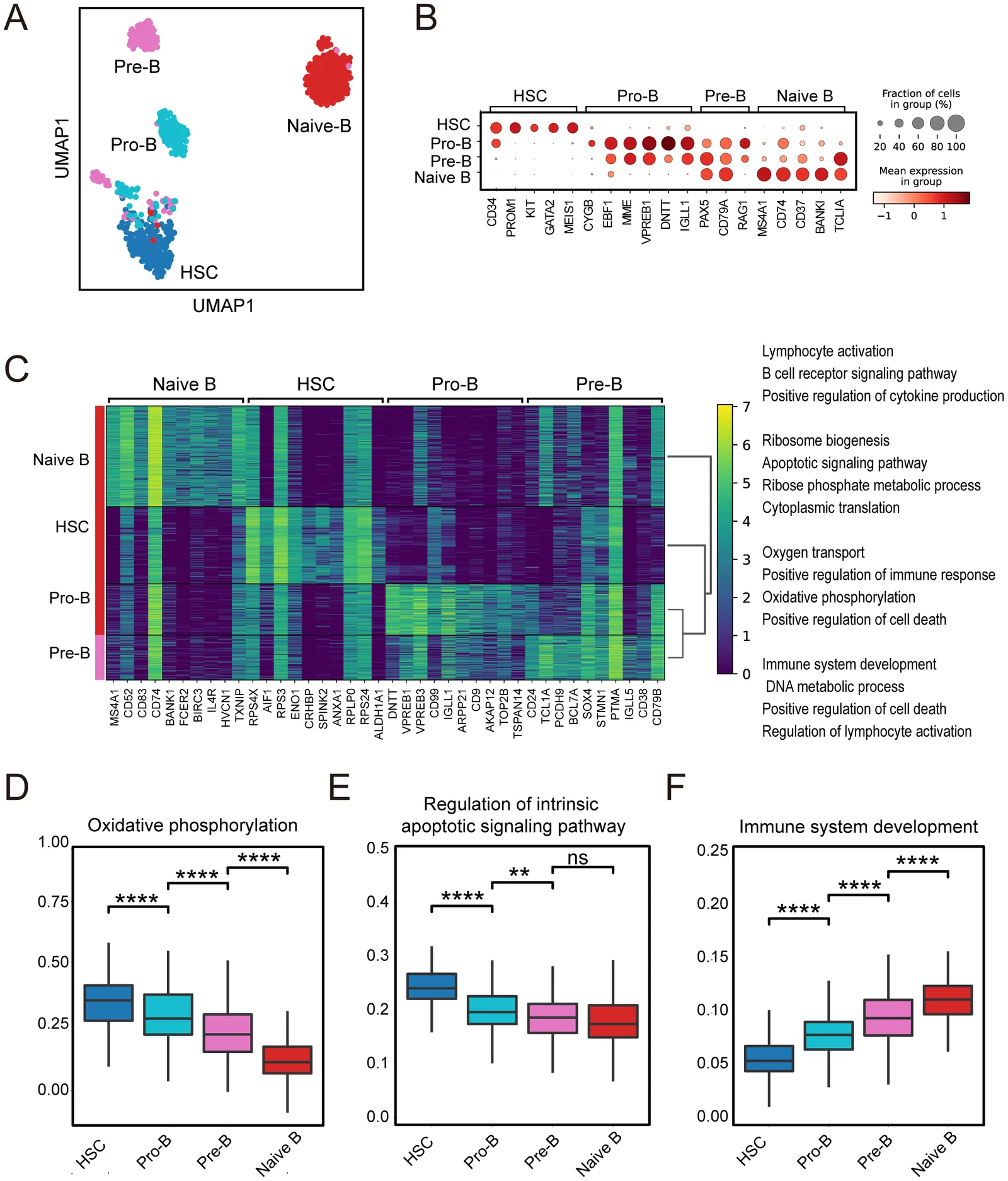
B cell differentiation analyzed by single-cell transcriptomic sequencing. **(A)** UMAP displays the transcriptional clusters for the four B cell populations. Colors correspond to cell clusters. **(B)** Dot plots show the relative expression of the top 5 signature genes for each cluster. **(C)** Heatmap shows the relative expression of the top 10 signature genes for each cluster. Significantly enriched biological processes for corresponding clusters are presented on the right. **(D–F)** Main expression patterns revealed by DEGs between HSC and differentiated B cells including **(D)** Oxidative phosphorylation (downregulated), **(E)** Regulation of intrinsic apoptotic signaling pathway (downregulated), **(F)** Immune system development (upregulated). The box plots illustrate the distribution patterns of module scores across clusters. Statistical comparisons between groups were performed using the Wilcoxon rank-sum test. Significance levels are indicated as follows: ns, not significant; *P < 0.05; **P < 0.01; ***P < 0.001; ****P < 0.0001.

To investigate whether Raman-observed biomolecular changes are reflected at the transcriptomic level, we analyzed published scRNA-seq datasets of HSC, pro-B, pre-B, and naive B cells (19). Consistent with previous reports, the transcriptomic data separated into four distinct phenotypes (**Figure 3A**), and the gene expression dot plot supports the reliability of our cell clustering (**Figure 3B**). The heatmap displays the relative expression of the top 10 signature genes for each cluster (**Figure 3C**). Metascape enrichment analysis revealed the signaling pathways highly expressed in each group (**Figure 3C**). Genes downregulated during differentiation were associated with oxidative phosphorylation and regulation of intrinsic apoptotic signaling pathway (**Figures 3D, E**). Furthermore, genes related to immune system development were more highly expressed in naive B cells than in other stages, suggesting that immune characteristics gradually emerge as HSCs differentiate into lymphoid cells.

### 3.4 Cross-modal alignment between Raman spectra and transcriptomic profiles

**Figure 4:**
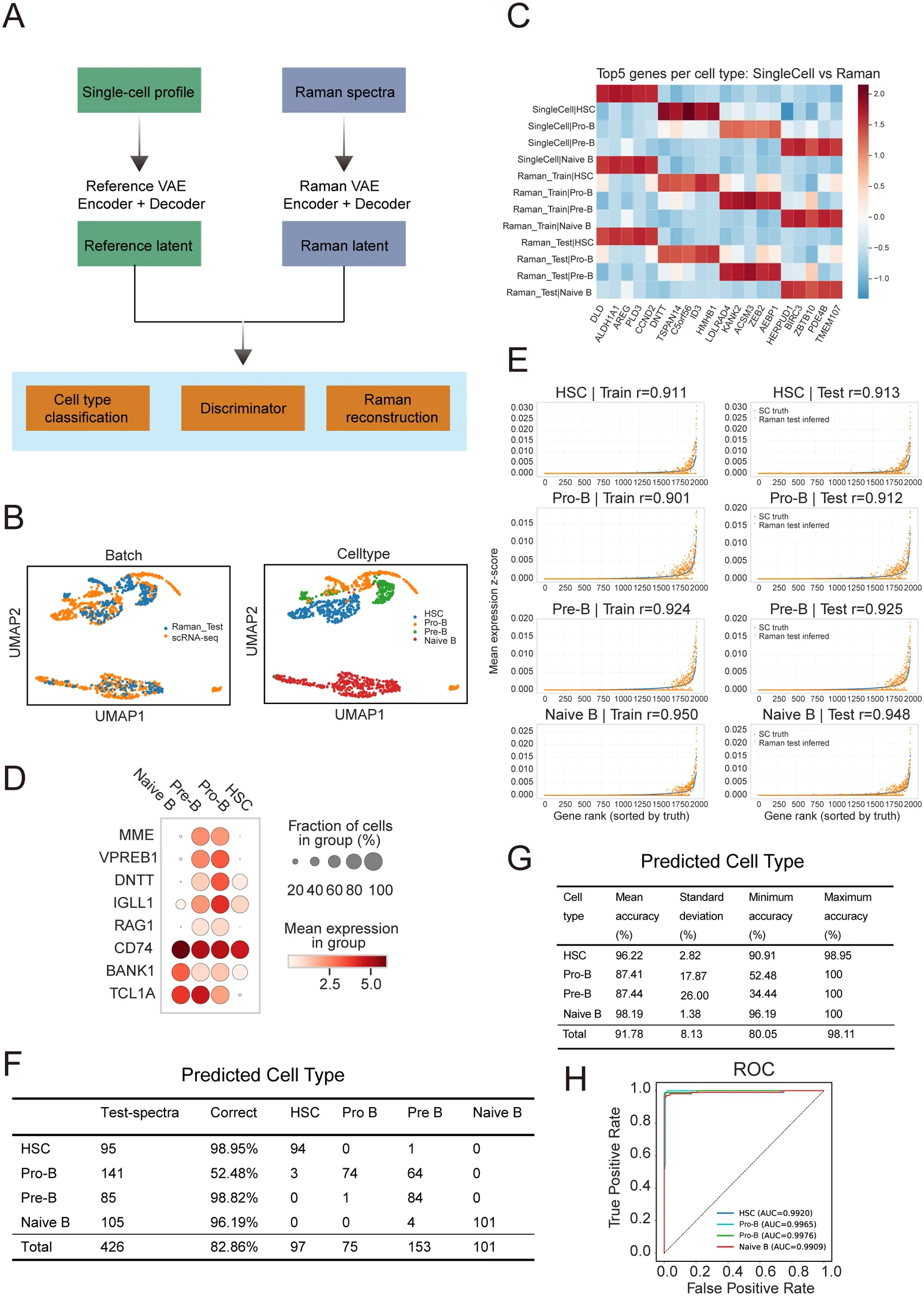
Cross-modal alignment between Raman spectra and transcriptomic profiles. **(A)** An adversarial autoencoder (AAE) model was used to transfer single-cell Raman measurements into a reference scRNA-seq expression space. Two autoencoders—one for Raman spectra and the other for scRNA-seq—are trained adversarially to align their latent representations into a shared, indistinguishable latent space. Once this common latent space is established, new Raman spectra are encoded using the Raman encoder, and the resulting latent embeddings are decoded into scRNA-seq profiles via the scRNA-seq decoder. This framework further enables subsequent cell type classification, discrimination, and Raman signal reconstruction. **(B)** UMAP visualization of AAE model–generated Raman-inferred and reference scRNA-seq profiles (left), with cells colored by cell type (right). **(C)** Heatmap showing the expression levels of the top five marker genes per cell type, as identified from both Raman-inferred and reference scRNA-seq profile datasets. Color intensity reflects gene expression magnitude, confirming strong consistency between the two modalities in capturing cell type–specific transcriptional signatures. **(D)** Comparison of representative marker genes between Raman-inferred and reference transcriptomic profiles. **(E)** Correlation analysis between Raman-inferred and reference transcriptomic profiles across HSC, pro-B, pre-B, and naive B cells. **(F)** Confusion matrix of AAE-based cell-type classification on the held-out test set. **(G)** Summary of AAE-based classification performance across repeated runs. Data are shown as mean accuracy ± SD for each cell type. **(H)** ROC curve analysis evaluating the classification performance of the integrated model. The curve demonstrates robust discrimination of cell types with a high area under the curve (AUC), validating the reliability of cell type annotations.

To investigate whether cellular Raman spectra could be aligned with cell-type-associated transcriptomic states during B-cell differentiation, we applied an AAE-based model to map Raman measurements into a reference scRNA-seq expression space (**Figure 4A**). UMAP visualization showed substantial overlap between Raman-inferred and reference scRNA-seq profiles while preserving the overall separation of major cell populations (**Figure 4B**). Expression patterns of representative marker genes were largely preserved between Raman-inferred and reference transcriptomic profiles (**Figures 4C, D**). Correlation analyses further demonstrated concordance between Raman-inferred and reference transcriptomic profiles across HSC, pro-B, pre-B and naive B cells (**Figure 4D**; **Supplementary Figures 6A–C**).

Model performance was evaluated using a cell-level held-out test set. Correlation analysis between Raman-inferred and reference transcriptomic profiles showed strong concordance across HSC, pro-B, pre-B, and naive B cells, with Pearson correlation coefficients exceeding 0.9 for all four cell types (**Figure 4E**). For cell-type classification, HSC, pre-B and naive B cells were classified with high accuracy (98.95%, 98.82% and 96.19%, respectively), whereas pro-B cells showed lower accuracy (52.48%), with most errors occurring between the pro-B and pre-B populations, suggesting that classification of these adjacent developmental stages is more sensitive to train-test partitioning. Overall, 353 of 426 test spectra were correctly classified, corresponding to an accuracy of 82.86% (**Figure 4F**). Repeated analyses using multiple random seeds and independent partitions showed that HSC and naive B cells remained relatively stable (mean accuracies of 96.22% ± 2.82% and 98.19% ± 1.38%, respectively), whereas pro-B and pre-B cells exhibited much wider ranges (52.48-100% and 34.44-100%, respectively) (**Figure 4G**). ROC analysis of the test set (**Figure 4H**) revealed near-perfect discrimination for all four cell types, with AUC values all exceeding 0.99. Additional baseline and control analyses showed that Raman-inferred profiles exceeded permuted-label controls and preserved marker-gene and pseudobulk differential-expression patterns, while predicted-cell-type mean-expression baselines performed similarly in the full 2,000-HVG space (**Supplementary Table 4**). This suggests that the current AAE framework primarily recovers cell-type-associated transcriptomic structure rather than providing substantial additional cell-specific information.

### 3.5 Raman spectroscopic discrimination of normal B cells and B-ALL cells

**Figure 5:**
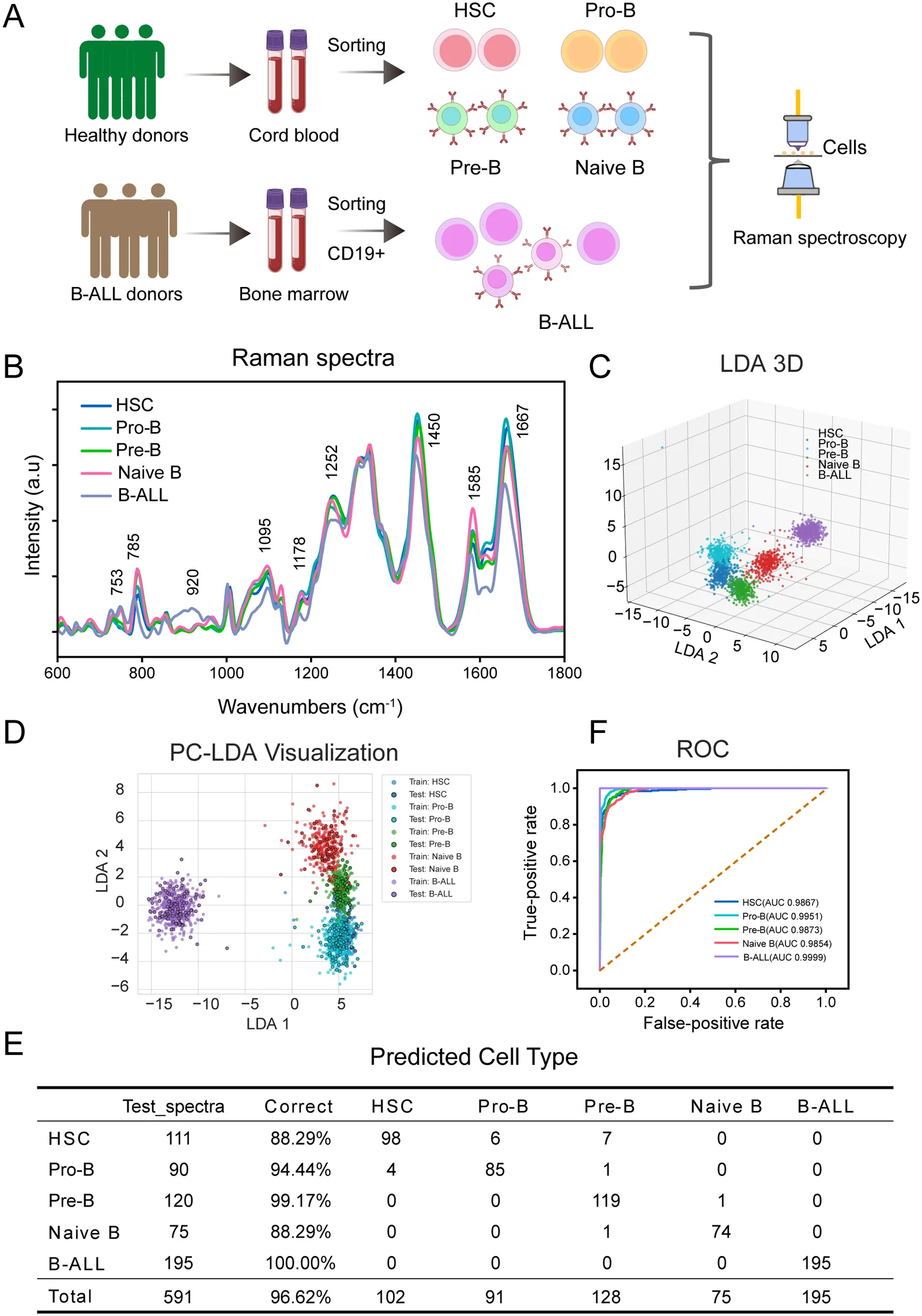
Comparison of normal and malignant B cells using Raman spectroscopy. **(A)** Schematic overview of the experimental workflow. Four differentiation stages of normal B cells were isolated from normal cord blood, and malignant B cells isolated from the bone marrow of B-ALL patients were analyzed by Raman spectroscopy. **(B)** Representative Raman spectra showing average spectral profiles of the five populations. Characteristic peak positions are labeled. **(C)** 3D LDA plot showing distinct clustering of the five populations based on Raman spectral features. Distinct colored clusters represent HSC, pro-B, pre-B, naive B, and B-ALL cells. **(D)** PC-LDA plot showing distinct clustering of the five populations based on Raman spectral features, indicating successful discrimination between normal and malignant B cells. Data points without black circles represent the training dataset, whereas those with black circles represent the test dataset. **(E)** Confusion matrix for the predicted classification of the five hematopoietic cell populations using the PC-LDA model. **(F)** ROC curves for the predicted classification of the five hematopoietic cell populations using the PC-LDA model. n = 544 spectra for B-ALL cells (3 donors per group).

B cells in B-ALL are heterogeneous, consisting predominantly of pro-B and pre-B cells, along with B cells at other developmental stages. We further compared the differences between normal B cells and B-ALL B cells (**Figure 5A**). A total of 1,957 Raman spectra were profiled to construct the Raman atlas. The averaged Raman spectra for each subtype are shown in **Figure 5B**, with characteristic bands at 753, 785, 920, 1095, 1178, 1252, 1450, 1585, and 1667 cm^-1^ playing key roles in separating the five cell populations. For visualization, a three-dimensional PC-LDA plot clearly distinguished the five cell populations (**Figure 5C**). The model trained on the training set achieved excellent separation of the five cell populations in a two-dimensional discriminant space (**Figure 5D**). Classification performance was evaluated on a cell-level held-out test set using a confusion matrix (**Figure 5E**), yielding an overall accuracy of 96.62%. Because this split was grouped by cell rather than by donor, cells from the same B-ALL patients and cord-blood donors may appear in both training and test sets; therefore, this 96.62% accuracy should be interpreted as an internal, within-cohort estimate rather than performance in previously unseen patients. The ROC curves on the test set (**Figure 5F**) demonstrated high discriminative ability within this internal split, with AUC values exceeding 0.98 for all five cell types.

### 3.6 Elevated glucose metabolism in B-ALL compared to normal B cells

**Figure 6:**
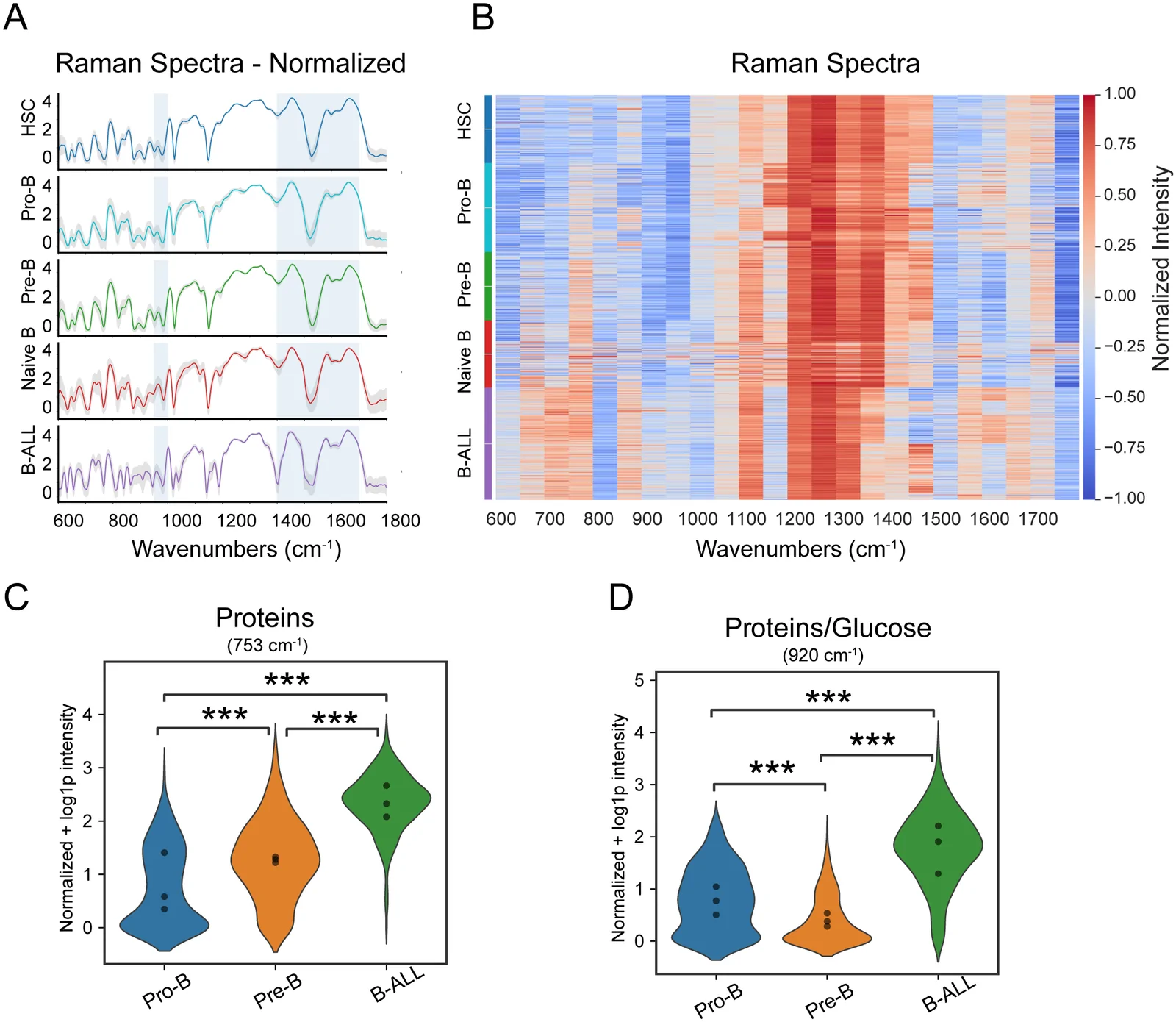
Raman spectroscopy analysis reveals differences in normal and malignant B cells. **(A)** Raman spectra of five hematopoietic cell populations (four normal B cell subsets and B-ALL cells), normalized to a maximum of 1 and scaled to zero mean and unit variance. **(B)** Heatmap of scaled Raman intensity across normal and malignant B cells. **(C, D)** Quantitative comparison of Raman band intensities for proteins (753 cm^-1^), and proteins/glucose (920 cm^-1^). Statistical comparisons between groups were performed using a linear mixed-effects model with cell population as a fixed effect and donor and cell (nested within donor) as random effects, accounting for the non-independence of repeated spectra from the same cell. Pairwise comparisons of estimated marginal means were FDR-adjusted (Benjamini–Hochberg). *P < 0.05; **P < 0.01; ***P < 0.001.

The scaled Raman average spectra are presented in **Figure 6A**. The shaded areas (950–1000 cm^-1^ and 1400–1700 cm^-1^) are the regions with the most pronounced differences. The heatmap in **Figure 6B** visualizes the scaled Raman intensity across the five cell subgroups in the 1100–1500 cm^-1^ range, showing that all cell types highly express these features, with no significant differences among them. However, in the 650–950 cm^-1^ range, a clear trend was observed: the Raman intensity in B-ALL cells was significantly higher than that in the four normal B cell types (**Figure 6B**). As shown in **Figures 6C, D**, the levels of protein and glucose metabolism-related features (753 and 920 cm^-1^) were markedly elevated in B-ALL cells compared to pro-B and pre-B cells. We also compared additional biomolecular features, including nucleic acids, lipids, and cytochrome c, as reflected by the Raman bands at 785, 1095, 1178, 1252, 1450, 1585, and 1667 cm^-1^ (**Supplementary Figures 7A–G**). The donor-level analysis results have been added as **Supplementary Figure 8**.

To determine whether the Raman spectral differences were accompanied by alterations in metabolism-related transcriptional programs, we performed targeted GSEA comparing B-ALL cells with normal B cells in the GSE48558 and GSE116229 datasets. HALLMARK_GLYCOLYSIS showed positive enrichment in B-ALL and met the FDR q < 0.25 criterion in both datasets (GSE48558: NES = 1.52, nominal P = 0.0017, FDR q = 0.028; GSE116229: NES = 1.74, nominal P < 0.001, FDR q = 0.019). GOBP_AEROBIC_RESPIRATION also showed positive enrichment and met the FDR criterion in both datasets, although nominal significance was dataset-dependent (GSE48558: NES = 1.18, nominal P = 0.128, FDR q = 0.206; GSE116229: NES = 1.49, nominal P = 0.0025, FDR q = 0.111). Representative enrichment plots are shown in **Supplementary Figures 9A–D**. Together, the Raman spectral differences and targeted GSEA results suggest alterations in glucose- and energy-metabolism-associated molecular features in B-ALL cells.

## 4 Discussion

In this study, we established a label-free, fixed-cell-based Raman spectroscopy platform for single-cell profiling of B cell differentiation and leukemic transformation, combined with AAE-based inference of transcriptomic profiles. Our results demonstrate that Raman spectral features—particularly those of cytochrome c, nucleic acids, proteins, and lipids—enable reliable discrimination of distinct B cell developmental stages with high classification accuracy (96.48%), and distinguish bone-marrow B-ALL from cord-blood-derived B cells as a proof-of-concept (96.62%). Beyond phenotypic classification, we show that Raman spectra can be used to infer transcriptomic features, revealing strong correlations between Raman-inferred and scRNA-seq expression across B cell developmental stages.

Although most scRNA-seq requires live or fresh-frozen cells, the technique is inherently destructive and fixed-sample data quality is often suboptimal. In contrast, our method is compatible with fixed cells, offering future potential for retrospective analysis of archived specimens. Previous studies have demonstrated that paraformaldehyde fixation preserves the Raman signature of cytochrome c (750 cm^-1^) and imposes minimal disruption to cellular molecular states (29, 30), and while fixation may cause some RNA degradation (31), the relative differences in cytochrome c and nucleic acid signals among cell types—the primary basis for our classification—are maintained. Our results demonstrated that the AAE-based model generated Raman-inferred profiles concordant with reference cell-type transcriptomic patterns. However, because Raman spectra and scRNA-seq profiles were not acquired from the same cells or matched samples, these results should be interpreted as cross-modal, reference-guided transcriptomic inference rather than direct single-cell transcriptome prediction. In the full 2,000-HVG space, the predicted-cell-type mean-expression baseline performed similarly to, and in some cases better than, the AAE output (**Supplementary Table 4**). This suggests that the current AAE framework primarily recovers cell-type-associated transcriptomic structure rather than providing substantial additional cell-specific information. Accordingly, our approach should be understood as reference-guided transcriptomic inference rather than a validated method for single-cell gene expression prediction. This extends the AAE-based model (1) from live mouse embryonic stem cells to fixed human hematopoietic cells, suggesting that fixation does not preclude transcriptomic inference. The ability to infer transcriptomic features from fixed-cell Raman spectra opens possibilities for retrospective multi-omic analysis of biobank specimens, where RNA is degraded but cellular biochemical signatures are preserved.

A limitation of this study is the tissue-source mismatch between the normal and leukemic cell populations. Normal B-cell differentiation stages were derived from umbilical cord blood, whereas B-ALL cells were obtained from adult bone marrow. Consequently, the observed spectral differences may partially reflect variations in tissue origin, donor age, or microenvironment, rather than disease status alone. Another methodological consideration concerns that the reference scRNA-seq data used for cross-modal alignment, which was derived from adult bone marrow while our Raman spectra were acquired from cord-blood-derived cells (19). However, Xie et al. have shown that cells of the same immunophenotypic type cluster together in transcriptomic space regardless of tissue origin, supporting the use of their adult bone marrow-derived dataset as a reference for aligning with our cord blood-derived Raman data (19). Nonetheless, future studies incorporating matched normal bone marrow B-cell progenitors and additional cord blood B-ALL samples processed under identical conditions will be essential to more rigorously disentangle disease-specific from tissue-specific contributions and to better assess the generalizability of our findings. A further limitation is that the reference scRNA-seq data are derived from two GEO accessions (GSE137864 for HSCs and GSE149938 for B-lineage cells) within the same Xie et al. atlas. Although both accessions come from a single integrated study, the HSC versus B-cell comparison cannot be fully disentangled from accession/tissue-source differences.

Our Raman and flow cytometry data revealed progressive changes in cytochrome c-associated Raman signals from HSCs to naive B cells, raising the question of whether this upregulation drives B cell differentiation or merely reflects increased metabolic demand. Cytochrome c is essential for mitochondrial electron transport and MMP generation. B cell differentiation is accompanied by increased MMP (**Figure 2E**) and mitochondrial biogenesis (32, 33), both requiring higher cytochrome c expression, suggesting that the observed increase likely reflects elevated metabolic demand rather than a differentiation-driving signal. At the same time, OXPHOS-associated transcriptional programs are downregulated during differentiation (**Figure 3D**). Although this appears contradictory, it reflects the distinction between protein-level mitochondrial function and transcriptional regulation of metabolic pathways (34). Increased MMP indicates enhanced mitochondrial function, whereas OXPHOS downregulation marks a metabolic shift toward glycolysis to support proliferation and effector functions (35). This metabolic remodeling is a hallmark of lymphocyte differentiation (34, 35). Cytochrome c upregulation also occurs during megakaryocytic differentiation without driving it (36), supporting that its accumulation is a consequence rather than a driver. Regardless, cytochrome c-associated Raman features serve as practical spectral markers for monitoring B cell differentiation status.

Unlike the Raman signals associated with cytochrome c and nucleic acids, our Raman analysis did not detect significant trends in protein- or lipid-associated spectral bands (1457 and 1667 cm^-1^) across B cell differentiation stages (**Figure 2C**). This negative finding is biologically informative. It suggests that the early phases of B cell development (from HSCs to naive B cells) are not accompanied by a global increase in protein or lipid content, despite substantial changes in mitochondrial activity and nucleic acid content. One interpretation is that the primary metabolic demands during this period are directed toward DNA replication and mitochondrial biogenesis, rather than general molecular expansion (34). This aligns with the observation that proliferating progenitor B cells (pro-B and pre-B) undergo rapid clonal expansion while maintaining relatively constant cellular protein and lipid concentrations, possibly through tightly regulated synthesis (34). Thus, the relative stability of protein and lipid Raman signals during the HSC-to-naive B transition highlights the specificity of metabolic reprogramming during early B cell development, with energy metabolism and nucleic acid synthesis earlier than global molecular accumulation.

Several limitations should be acknowledged. First, although we performed donor-level analyses to assess inter-donor variability, the limited number of donors (n = 3 per group) reduced the statistical power of donor-level comparisons, highlighting the need for validation in larger cohorts. Second, our Raman measurements were performed on fixed cells; whether the observed spectral features fully recapitulate live-cell dynamics requires further investigation. Third, while we have interpreted the 1585 cm^-1^ band as a cytochrome c-associated feature based on its co-variation with other related bands, Raman spectroscopy alone cannot unambiguously distinguish cytochrome c from nucleic acid contributions at this wavenumber. Future studies employing resonance Raman or correlative immunofluorescence would be required to confirm its molecular origin definitively. Fourth, the flow cytometry validation was performed on broadly defined HSPCs and total B cells rather than the four individual stages resolved by Raman spectroscopy; thus, it validates a broad difference between the two extremes rather than the complete sequential pattern. Future studies using sorted intermediate populations will be required to validate the full differentiation trajectory at the protein level. Finally, future work could extend this platform to incorporate other single-cell modalities, such as scATAC-seq or metabolomics, and validate the approach in larger clinical cohorts. Despite these limitations, this study establishes Raman spectroscopy, integrated with machine learning and transcriptomic alignment, as a powerful tool for non-destructive fixed-cell omic profiling, with potential implications for future hematopoietic research and clinical exploration.

## Data Availability

The complete analysis code, AAE model configuration, random seeds, grouped train/test assignment scripts, and validation notebooks are publicly available at https://github.com/Bio-MingChen/Raman2SC-AAE. The repository includes dedicated documentation describing the reference scRNA-seq object, Raman preprocessing, model architecture, training settings, repeated random seeds, grouped train/test assignments, and software/package versions. Raw and processed Raman spectra, cell and donor metadata, preprocessing scripts, model parameters, and complete GSEA outputs are available from the corresponding author upon reasonable request, as the file sizes exceed GitHub’s storage limits.
