# Supplementary material for: Single-cell Raman profiling of B cell differentiation and leukemic transformation with transcriptome inference
Source: Front Immunol. 2026 Aug 27;17:1926870. doi: 10.3389/fimmu.2026.1926870 (PMC13557991; doi:10.3389/fimmu.2026.1926870)
Supplement: Supplementary file 1 [file Supplementaryfile1.docx]

Supplementary Material

# 1 Supplementary Figures and Tables

## 1.1 Supplementary Figures

**Supplementary Figure 1.** Gating strategy for flow cytometry analysis.(A)  FSC‑A vs SSC‑A for lymphocyte gating; (B) Dead cell exclusion (DAPI^-^) to retain only viable cells; (C) FSC‑A vs FSC‑H to exclude doublets/aggregates.

**Supplementary Figure 2.** Post-sort purity assessment of B cell developmental subsets (HSC, pro-B, pre-B, and naive B cells). The purity of sorted cell populations was verified by flow cytometry and exceeded 98% for all populations.

**Supplementary Figure 3.** **Spectral preprocessing workflow.** (A) Raw Raman spectra, showing inherent noise and baseline drift. (B) Spectra after smoothing, which reduces high-frequency noise while preserving characteristic spectral peaks. (C) Spectra after baseline correction and normalization, eliminating baseline offset and standardizing signal intensity for subsequent multivariate analysis.

**Supplementary Figure 4.** Dimensionality reduction visualization of B cells at four differentiation stages. (A) 2D projection of LDA1 vs LDA2, (B) 2D projection of LDA1 vs LDA3, (C) 3D LDA visualization, and (D) UMAP embedding derived from LDA-transformed data, demonstrating clear separation of sequential B cell developmental stages.

**Supplementary Figure 5.** Quantitative analysis of characteristic Raman peak intensities corresponding to intracellular biomolecules across four cell groups.(A-G) Bar plots showing the normalized Raman band intensity of Cytochrome c, nucleic acids, lipids/phospholipids and proteins at specific wavenumbers: (A) Cytochrome c at 746 cm^-1^; (B) Nucleic acid at 785 cm^-1^; (C) Nucleic acid at 966 cm^-1^; (D) Cytochrome c at 1124 cm^-1^; (E) Lipids/Phospholipids at 1457 cm^-1^; (F) Cytochrome C and nucleic acid composite peak at 1585 cm^-1^; (G) Proteins/Lipids at 1667 cm^-1^. Four experimental groups: HSC (green), Pro-B (orange), Pre-B (light blue), Naive B (purple). All data are presented as mean ± standard error of the mean (SEM). Statistical comparisons between groups were performed using one-way ANOVA followed by Tukey’s HSD post-hoc test. ns, not significant; *P < 0.05; **P < 0.01.

**Supplementary Figure 6.** Validation of Raman-inferred gene expression in B cell differentiation.(A-B) Pseudo-bulk gene proportion plots comparing scRNA-seq reference expression with Raman-inferred gene expression for the training and test sets, showing high concordance in global gene expression distribution. (C) Bar plot of Pearson correlation coefficients between Raman-inferred and true mean expression across four cell populations: HSC, pro-B, pre-B, and naive B cells, all exceeding 0.9.

**Supplementary Figure 7.** Quantitative comparison of Raman band intensities for (A) nucleic acid (785 cm^-1^), (B) lipids (1095 cm^-1^), (C) nucleic acid (1178 cm^-1^), (D) nucleic acid (1252 cm^-1^), (E) lipids/phospholipids and proteins (1450 cm^-1^), (F) cytochrome c and nucleic acid (1585 cm^-1^), and (G) proteins/lipids (1667 cm^-1^). Statistical comparisons between pro-B, pre-B, and B-ALL cells were performed using a linear mixed-effects model with cell population as a fixed effect and donor and cell (nested within donor) as random effects. Pairwise comparisons of estimated marginal means were FDR-adjusted (Benjamini–Hochberg). *P < 0.05; **P < 0.01; ***P < 0.001. Full results are provided in Supplementary Table 2.

**Supplementary Figure 8.** Quantitative comparison of characteristic Raman peak intensities of biomolecules in Pro-B, Pre-B and B-ALL cell groups. (A-I) Bar graphs display normalized Raman band intensities at distinct wavenumbers assigned to different intracellular biomolecules: (A) 753 cm^-1^, Proteins; (B) 826 cm^-1^, Nucleic acid; (C) 850 cm^-1^, Proteins/Glucose; (D) 1096 cm^-1^, Lipids; (E) 1178 cm^-1^, Nucleic acid; (F) 1232 cm^-1^, Nucleic acid; (G) 1460 cm^-1^, Lipids/Phospholipids/Proteins; (H) 1585 cm^-1^, Cytochrome C/Nucleic acid; (I) 1667 cm^-1^, Proteins/Lipids. Three cell groups: Pro-B (red), Pre-B (green), B-ALL (purple). All data are expressed as mean ± standard error of the mean (SEM). Intergroup statistical differences were evaluated by one-way ANOVA followed by Tukey’s HSD post-hoc multiple comparison test. ns, not significant; *P < 0.05; **P < 0.01; ***P < 0.001.

**Supplementary Figure 9.** Targeted GSEA of glycolysis and aerobic respiration in two independent B-ALL transcriptomic datasets. Gene set enrichment analysis (GSEA) was performed to compare B-ALL cells with normal B cells in GSE48558 (A-B) and GSE116229 (C-D). The gene sets analyzed were HALLMARK_GLYCOLYSIS (A-B), and GOBP_AEROBIC_RESPIRATION (C-D). The normalized enrichment score (NES), nominal p value, and false discovery rate (FDR) q value are shown in each panel. Gene-set enrichment was considered statistically significant when FDR q < 0.25. Nominal p values are shown for descriptive purposes only.

## 1.2 Supplementary Figures

**Supplemental Table 1.** Characteristics of selected leukemia patients.

| **Gender** | **Age** | **Genetic feature** | **Morphologic Subtype** | **Bast percentages** | **Immune phenotype** |
| --- | --- | --- | --- | --- | --- |
| M | 29 | BCR::ABLP190+,t(9;22) | B-ALL (Ph+) | 98.5% | CD19^+^CD10^+^CD34^+^ |
| M | 25 | EP300::ZNF384 | B-ALL (Ph-) | 97.2% | CD19^+^CD10^+^CD34^+^ |
| F | 30 | MLL::AF4(+), | B-ALL (Ph-) | 99.4% | CD19^+^CD10^+^CD34^+^ |

B-ALL: B-Acute Lymphoblastic Leukemia.
